# Dietary glycemic load and gastric cancer risk in Italy

**DOI:** 10.1038/sj.bjc.6604894

**Published:** 2009-02-03

**Authors:** P Bertuccio, D Praud, L Chatenoud, E Lucenteforte, C Bosetti, C Pelucchi, M Rossi, E Negri, C La Vecchia

**Affiliations:** 1 Istituto di Ricerche Farmacologiche ‘Mario Negri’, Via Giuseppe La Masa, 19, 20156 Milan, Italy; 2 Institut d'Informatique et de Mathématiques Appliquées, Université Joseph Fourier, BP 53, 38041 Grenoble Cedex 9, France; 3 Istituto di Statistica Medica e Biometria ‘Giulio A. Maccacaro’, Università degli Studi di Milano, 20133 Milan, Italy

**Keywords:** case-control studies, dietary glycemic load, glycemic index, gastric cancer

## Abstract

We investigated gastric cancer risk in relation to dietary glycemic index (GI) and glycemic load (GL), which represent indirect measures of carbohydrate absorption and consequently of dietary insulin demand, in a case-control study conducted in northern Italy between 1997 and 2007, including 230 patients with the incident, histologically confirmed gastric cancer and 547 frequency matched controls, admitted to the same hospitals as cases with acute non-neoplastic conditions. We used conditional logistic regression models, including terms for major recognised gastric cancer risk factors and non-carbohydrate energy intake. The odds ratios (ORs) in the highest *vs* lowest quintile were 1.9 (95% CI: 1.0–3.3) for GI and 2.5 (95% CI: 1.3–4.9) for GL. Compared with participants reporting low GL and high fruits/vegetables intake, the OR rose across strata of high GL and low fruits/vegetables, to reach 5.0 (95% CI: 2.2–11.5) for those reporting low fruits/vegetables intake and high GL. Our study may help to explain the direct relation observed in several studies between starchy foods and gastric cancer risk.

A direct relation between starchy foods – particularly refined cereals – and gastric cancer has long been observed in studies conducted in Greece (Trichopoulos *et al*, 1985), Italy (La Vecchia *et al*, 1987) and Belgium (Tuyns *et al*, 1992), and has been confirmed in most subsequent studies (World Cancer Research Fund/American Institute for Cancer Research, 2007; Lucenteforte *et al*, 2008; Navarro Silvera *et al*, 2008). Refined cereals and starchy foods, particularly in southern Europe, considered indicators of a poorer diet, have been associated with increased gastric cancer risk (La Vecchia and Franceschi, 2000); they may be associated with a diet high in glycemic index (GI) and glycemic load (GL).

The GI is an indicator of the absorption rate of carbohydrates and ranks their effect on blood glucose concentrations. It compares available carbohydrates gram-for-gram in individual foods, providing a numerical, evidence-based index of postprandial glycemia (Jenkins *et al*, 1981, 1984; Gnagnarella *et al*, 2004). As the GL combines measures of carbohydrate (both qualitative and quantitative) and of dietary insulin demand, the overall GI reflects the average quality of carbohydrates consumed, whereas the total dietary GL reflects both their average quantity and quality (Foster-Powell *et al*, 2002).

GI and, particularly, GL have been related to excess risk of colorectal (Augustin *et al*, 2002), breast (Augustin *et al*, 2001), oral and oesophageal (Augustin *et al*, 2003) cancers. Gastric cancer has also been associated with GI and GL in a case-control study in Italy (Augustin *et al*, 2004), though not in a Swedish cohort study (Larsson *et al*, 2006). We have further considered the relation between GL, GI and gastric cancer risk using data from another Italian case-control study, based on a more detailed, reproducible and validated food frequency questionnaire (FFQ) (Lucenteforte *et al*, 2008).

## Materials and methods

We derived data from a case-control study of gastric cancer conducted in 1997–2007 in the greater Milan area, Italy, the design of which has been described earlier (Lucenteforte *et al*, 2008). Briefly, cases were 230 patients (143 men, 87 women; median age 63 years, range 22–80 years) admitted to major teaching and general hospitals in the study area with the incident, histologically confirmed stomach cancer (ICD IX 151.0–151.9), diagnosed no longer than 1 year before the interview, and with no earlier diagnosis of cancer. The control group included 547 patients (286 men, 261 women; median age 63 years, range 22–80 years) frequency matched to cases by age and sex (ratio of 2 : 1 for men and 3 : 1 for women), admitted to the same hospitals as cases for a wide spectrum of acute, non-neoplastic conditions, unrelated to risk factors for stomach cancer and long-term diet modification. Of these 20% were admitted for traumas, 23% for other orthopaedic conditions, 22% for acute surgical and 35% for other miscellaneous disorders. Less than 5% of cases and controls who were approached refused to be interviewed.

For both cases and controls, data were collected during their hospital stay by trained interviewers using a structured questionnaire covering socio-demographic characteristics, anthropometric measures, selected lifestyle habits, including tobacco and alcohol consumption, personal medical and family history of cancer. The patients' usual diet during the two years before diagnosis or hospital admission (for controls) was assessed through a reproducible (Franceschi *et al*, 1995) and valid (Decarli *et al*, 1996) FFQ covering 78 foods and beverages, including a range of the most common recipes in Italian diet. Participants indicated the average weekly frequency of consumption for each dietary item; intakes lower than once a week but at least once a month were coded as 0.5 per week. Energy and carbohydrate intake were computed from the FFQ using an Italian food composition database, integrated with other sources when needed (Salvini *et al*, 1998; Gnagnarella *et al*, 2004).

GI values were assigned to each food item using international tables (Foster-Powell *et al*, 2002). Daily average GI was calculated by summing the products of the carbohydrate content per serving for each food or food group, times the average number of servings of that food per week, times its GI (Wolever *et al*, 1994), all divided by the total amount of available carbohydrates. The daily average GL was computed as the GI, but without dividing by the total amount of carbohydrates.

Odds ratios (ORs) and their corresponding 95% confidence intervals (CIs) for subsequent quintiles of GI and GL were estimated using conditional multiple logistic regression models, conditioned on age and sex (Breslow and Day, 1980). We considered two models: the first included terms for period of interview, education, body mass index, tobacco smoking, intake of fruits and vegetables, and family history of stomach cancer; the second also included non-carbohydrate energy intake, to allow for bias due to systematic over- or under-reporting (Willett and Stampfer, 1986).

## Results

Table 1 gives the distribution of gastric cancer cases and controls according to quintiles of GI and GL, and the corresponding multivariate ORs. The OR for the highest *vs* the lowest quintile of GI was 2.1 (95% CI: 1.2–3.6), with a significant trend in risk (*P*=0.034). For GL, ORs were significantly above unity in the third (OR=2.5, 95% CI: 1.4–4.5), fourth (OR=2.7, 95% CI: 1.5–4.9) and highest quintile (OR=2.7; 95% CI: 1.5–4.8), with a significant trend in risk (*P*<0.001). Continuous ORs for the difference between the 80th and 20th percentile (based on the control distribution) were 1.4 (95% CI: 1.1–1.9) for GI and 1.5 (95% CI: 1.2–2.0) for GL. After further adjustment for non-carbohydrate energy intake, the OR estimates were similar for both GI and GL.

Table 2 gives the continuous ORs of gastric cancer for GI and GL in strata of sex, age and major established risk factors for gastric cancer. No significant differences emerged in any strata of the covariates considered.

We further considered the combined effect of fruits/vegetables intake and GL on gastric cancer risk. Compared with participants reporting low GL and high fruits and vegetables intake, the OR rose for increasing levels of GL, particularly in participants with low fruits/vegetables intake, to reach 5.0 (95% CI: 2.2–11.5) for those reporting high GL and low fruits/vegetables intake.

## Discussion

This study, based on a valid and detailed FFQ, confirms and further quantifies the existence of a direct relation between GI, and mostly GL, and gastric cancer risk. We were able to allow in the analysis not only for major risk factors for gastric cancer, such as education, tobacco smoking and vegetables and fruits intake, but also for non-carbohydrate energy intake. Out results indicate that some characteristics of carbohydrates, in particular of such refined carbohydrates as white bread, pasta and rice, have an appreciable influence on gastric cancer risk in this population.

The findings are consistent with those of an earlier Italian case-control study, based on a shorter questionnaire, which found an approximately two-fold risk of gastric cancer for the highest quartile of GL, and only a modest association with GI (Augustin *et al*, 2004). They also agree with several earlier studies, indicating that a diet rich in cereals or starches is associated with an increased gastric cancer risk (Trichopoulos *et al*, 1985; La Vecchia *et al*, 1987; Tuyns *et al*, 1992; World Cancer Research Fund/American Institute for Cancer Research, 2007; Lucenteforte *et al*, 2008; Navarro Silvera *et al*, 2008). They are, however, at variance with the findings of the Swedish Mammography Cohort (Larsson *et al*, 2006), which found no relation between GI and GL and gastric cancer risk.

We also found that the role of GL is independent of other major dietary correlates for gastric cancer, including vegetables and fruits consumption, and that there was a five-fold difference in gastric cancer risk between participants reporting low GL and high fruits and vegetables intake and those reporting high GL and low fruits and vegetables consumption. Thus, two simple diet indicators determine substantial variation in gastric cancer risk.

A high GL diet may increase gastric cancer risk through the modulation of the insulin-like growth factors (IGFs). Insulin increases the activity of IGFs, such as IGF-1 (Gnagnarella *et al*, 2008), and suppresses hepatic secretion of IGF-binding protein-1. IGF-1 stimulates cell proliferation and differentiation, inhibits apoptosis (Kaaks and Lukanova, 2001; Gnagnarella *et al*, 2008) and increases production of vascular endothelial growth factors, important in tumour angiogenesis, and the expression of glucose transporters, glycolytic enzymes, and growth factors, which may promote tumor cell survival under hypoxic conditions (Akakura *et al*, 2001; Oh *et al*, 2008). Higher IGF-1 concentrations have been observed in patients with gastric cancer than in healthy controls (Franciosi *et al*, 2003). Insulin can also influence sex hormone concentrations and reduce the levels of their binding protein (Garzo and Dorrington, 1984; Poretsky and Kalin, 1987; Gnagnarella *et al*, 2008). Sex hormones have been linked to gastric cancer risk (La Vecchia *et al*, 1994), though this has been disputed (Bahmanyar *et al*, 2008; Persson *et al*, 2008).

A limitation of our study is the lack of information on *Helicobacter pylori* (*H. pylori*) infection. A recent study observed a positive association between plasma glucose concentration and risk of stomach cancer only among *H. pylori*-positive participants (Yamagata *et al*, 2005; Larsson *et al*, 2006). Though the prevalence of *H. pylori* infection is declining (Malaty, 2007), it was relatively high (about 45%) in Italy in the mid 1990's. It increased with age and was more frequent in men than in women (Russo *et al*, 1999). Thus, a large proportion of the population studied is likely to be *H. Pylori*-positive.

Participation among eligible patients was almost complete, and the catchment areas for cases and controls were comparable. Hospital controls may differ in their dietary habits from the general population. However, we excluded control participants admitted for conditions associated with dietary modifications (diabetes mellitus, cardiovascular diseases, etc.). Recall bias is also unlikely, given that the association between various types of starches and gastric cancer was not evident at the time of interviews. Moreover, the similar hospital setting for cases and controls likely increased the comparability of dietary histories, and the questionnaire was reproducible and valid (Franceschi *et al*, 1995; Decarli *et al*, 1996; D'Avanzo *et al*, 1997).

With respect to confounding results, we adjusted for non-carbohydrate energy intake to control for potential systematic over- or under-reporting between cases and controls (Willett and Stampfer, 1986); further adjustment for several covariates failed to explain the association between GI and GL and gastric cancer.

Our study suggests that a diet high in GL and GI is directly related to gastric cancer risk, and may therefore help to explain the relation observed in several studies with starchy foods (Trichopoulos *et al*, 1985; La Vecchia *et al*, 1987; Buiatti *et al*, 1990; Tuyns *et al*, 1992).

## Figures and Tables

**Table 1:** Odds ratios (ORs) and 95% confidence interval (CI) of gastric cancer among 230 cases and 547 controls, according to glycemic index and glycemic load. Italy, 1997–2007

	**Upper limit**	**Cases *n* (%)**	**Controls *n* (%)**	**OR^a^ (95% CI)**	**OR^b^ (95% CI)**
*Glycemic index*					
I (low)	73	28 (12.2)	110 (20.1)	1.0^c^	1.0^c^
II	77	44 (19.1)	109 (19.9)	1.5 (0.9–2.7)	1.5 (0.9–2.6)
III	80	44 (19.1)	110 (20.1)	1.5 (0.8–2.6)	1.4 (0.8–2.4)
IV	83	47 (20.4)	108 (19.7)	1.5 (0.8–2.6)	1.4 (0.8–2.5)
V (high)	—	67 (29.1)	110 (20.1)	2.1 (1.2–3.6)	1.9 (1.0–3.3)
*χ*_1_^2^ trend (*P*-value)				4.5 (*P*-value=0.034)	3.0 (*P*-value=0.083)
Continuous^d^				1.4 (1.1–1.9)	1.4 (1.0–1.9)
					
*Glycemic load*					
I (low)	147	24 (10.4)	109 (19.9)	1.0^c^	1.0^c^
II	183	30 (13.0)	110 (20.1)	1.4 (0.7–2.5)	1.3 (0.7–2.5)
III	217	57 (24.8)	110 (20.1)	2.5 (1.4–4.5)	2.4 (1.3–4.5)
IV	263	58 (25.2)	108 (19.7)	2.7 (1.5–4.9)	2.6 (1.4–5.1)
V (high)	—	61 (26.5)	110 (20.1)	2.7 (1.5–4.8)	2.5 (1.3–4.9)
*χ*_1_^2^ trend (*P*-value)				13.4 (*P*-value<0.001)	8.9 (*P*-value=0.003)
Continuous^d^				1.5 (1.2–2.0)	1.4 (1.1–1.9)

a Estimates from conditional logistic regression models, conditioned on age and sex and adjusted for period of interview, only when indicated, education, body mass index, tobacco smoking, intake of fruits and vegetables, and family history of stomach cancer.

b Further adjusted for non-carbohydrate energy intake.

c Reference category.

d The unit is the difference between the 80th and the 20th percentile.

**Table 2:** Odds ratios (ORs) and 95% confidence intervals (CI) of gastric cancer among 230 cases and 547 controls, according to glycemic index and glycemic load in strata of selected covariates. Italy, 1997–2007

**Covariates**	**OR (95 % CI)^a^^,^^b^**	
**(no. of cases/no. of controls)**	**Glycemic index**	**Glycemic load**
*Sex*		
Men (143/286)	1.1 (0.7–1.6)	1.5 (1.0–2.1)
Women (87/261)	1.8 (1.1–2.9)	1.3 (0.8–2.3)
		
*Age (years)*		
<65 (125/297)	1.5 (1.0–2.3)	1.3 (0.9–2.0)
⩾65 (105/250)	1.3 (0.8–2.1)	1.7 (1.1–2.7)
		
*Education*^c^ *(years)*		
<7 (95/236 )	1.7 (1.0–3.0)	1.4 (0.9–2.4)
⩾7 (132/307)	1.1 (0.8–1.6)	1.4 (0.9–2.0)
		
*Body mass index*^c^ *(kg m*^*−2*^*)*		
<25 (118/248)	1.2 (0.8–1.8)	1.3 (0.8–2.0)
⩾25 (108/297)	1.6 (1.0–2.6)	1.6 (1.0–2.4)
		
*Smoking status* c		
Non-smoker (171/428)	1.5 (1.0–2.2)	1.3 (0.9–1.8)
Smoker (58/118)	1.2 (0.6–2.3)	2.1 (1.1–3.9)
		
*Intake of fruits (portions/week)*		
⩽15 (117/273)	1.5 (1.0–2.3)	1.5 (1.0–2.2)
>15 (113/274)	1.6 (1.0–2.5)	1.4 (0.9–2.2)
		
*Intake of vegetables (portions/week)*		
⩽9 (141/274)	1.4 (0.9–2.1)	1.3 (0.9–2.0)
>9 (89/273 )	1.2 (0.8–1.9)	1.4 (0.9–2.2)

a The unit is the difference between the 80th and the 20th percentile.

b Estimates from conditional logistic regression models, conditioned on age and sex and adjusted for period of interview, only when indicated, education, body mass index, tobacco smoking, intake of fruits and vegetables, family history of stomach cancer and non-carbohydrate energy intake.

c The sum does not add up to the total because of some missing values.
